# Associations between household characteristics and environmentally persistent free radicals in house dust from two Australian locations

**DOI:** 10.3389/fpubh.2025.1603114

**Published:** 2025-06-18

**Authors:** Wen Ray Lee, Prakash Dangal, Gaurav Langan, Nina Lazarevic, Zhiwei Xu, Stephania A. Cormier, Slawo Lomnicki, Peter D. Sly, Dwan Vilcins

**Affiliations:** ^1^Children’s Health and Environment Program, Child Health Research Centre, The University of Queensland, Brisbane, QLD, Australia; ^2^Superfund Research Centre, Louisiana State University, Baton Rouge, LA, United States; ^3^National Centre for Epidemiology and Population Health, Australian National University, Canberra, ACT, Australia; ^4^School of Medicine and Dentistry, Griffith University, Gold Coast, QLD, Australia

**Keywords:** environmentally persistent free radicals, EPFRs, indoor air pollution, particulate matter, house dust

## Abstract

**Introduction:**

The association between air pollution and adverse health outcomes has been extensively documented, with oxidative stress widely considered a contributing factor. However, the precise underlying mechanism(s) remains unclear. Recent studies suggest that environmentally persistent free radicals (EPFRs) may provide the missing connection between air pollution and its detrimental health effects. Nevertheless, the indoor environment has received limited attention in EPFR research. Therefore, in this study, we measured EPFRs in house dust samples from two locations in Australia and examined household characteristics associated with their presence.

**Methods:**

Household characteristics and behaviours that influence indoor air quality were collected from an online questionnaire; 24-h indoor and outdoor air quality were measured with a TSI DustTrak™ DRX Aerosol monitor 8,533; annual indoor and outdoor air quality were matched to two validated, satellite-based land-use regression models; and dust samples were collected from house vacuums. Dust samples were analyzed using nano electron paramagnetic resonance (EPR) to determine spin concentrations, g-factor, and delta H peak-to-peak (Hp-p). Key variables were identified using Lasso-penalized regression models, followed by unpenalized linear regression and post-selection inference to estimate coefficients and assess the robustness of the findings.

**Results:**

Our analysis revealed that factors such as extractor fan usage during cooking, exposure to traffic-related air pollution and ambient PM_2.5_ levels, indoor combustion activities, seasonal variation, housing construction type, ventilation, and cleaning practices were significantly associated with EPFR concentrations in Australian homes. Notably, consistent use of extractor fans during cooking was strongly and consistently associated with lower EPFR concentrations in house dust across both study locations.

**Discussion:**

Our research provided insight into the potential impact of household characteristics on EPFR concentrations, which can potentially lead to adverse health effects. Future research should link our research findings on factors affecting indoor EPFRs to their potential health effects.

## 1 Introduction

The association between air pollution and adverse health outcomes has been long established, but the causal mechanism(s) behind this association are still uncertain (1–4). Recent studies propose that an oxidant component of particle air pollution, environmentally persistent free radicals (EPFRs), may serve as an inducer of oxidative stress (OS), potentially bridging the gap between air pollution and its detrimental health impacts (5–14). EPFRs are formed during combustion processes and are typically associated with fine particulate matter (PM_2.5_; particles with a mass median aerodynamic diameter of ≤2.5 μm). These radicals are commonly produced from sources such as traffic-related air pollution (TRAP), residential combustion activities, cooking, industrial burning, cigarette smoking/vaping, and wildfires (6, 10–12, 14–17). EPFRs are particularly concerning as they are free radicals that can persist in the environment and biological systems for prolonged periods (8, 10–12, 14). Typically, free radicals have short lifetimes—only a few picoseconds—due to the extreme instability of unpaired electron/s in their outermost orbit (8, 11); however, EPFRs are unusually stable and long-lasting. This stability arises from the continuous cycling of their valence electrons between paired and unpaired states, facilitated by electron transfer from organic particles to the surfaces of redox-active transition metals present in particulate matter (PM) (6, 7). As a result, these stabilized EFPRs can persist for extended periods, lasting up to 5 years in certain environments, and are notably resistant to decomposition (8, 12, 14).

Little is known about the presence of EPFRs in the household environment. Studies that have explored household environments mostly focused on airborne EPFRs produced from solid fuel combustion used for cooking and heating, demonstrating household practices can be important sources for residential EPFRs (13, 14, 18–20). In addition, motor vehicle emissions, a major source of ambient EPFRs, have been extensively reviewed (16, 21, 22). Household practices such as air-conditioning and window opening can influence TRAP penetration into indoor environments (23). Consequently, it is believed that EPFRs generated from TRAP can accumulate in homes as house dust through the infiltration of ambient air. Emerging evidence shows the potential for adverse health effects of household EPFRs (10), particularly for children, due to their increased sensitivity to exposures and continuing respiratory development (24). Sly et al. reported that 89 out of 90 house dust samples in Brisbane, Australia, had detectable EPFRs; high concentrations (≥ 6 × 10^17^ spins/g) were associated with an increased risk of wheezing in young children compared to low EPFR concentrations (< 4 × 10^17^ spins/g) (10). The health concerns from EPFR exposure highlight the importance of understanding the factors that influence the presence of EPFRs in indoor environments, particularly in residential homes. We hypothesize that household characteristics and behaviors influence EPFR concentration in house dust. This study aimed to determine household characteristics associated with EPFRs in Australian homes.

## 2 Materials and methods

### 2.1 Population

The data used in the present study were collected in two cohort studies in Australia: the Early Life Lung Function (ELLF) study and the Barwon Infant Study (BIS).

The ELLF study is a longitudinal birth cohort from Brisbane, Australia, enrolled during pregnancy and followed until age 7. Brisbane is located in Southeast Queensland, and this city has a subtropical climate with average temperatures ranging from 21°C to 30.4°C during summer and 10.5°C to 23.4°C in winter (25). The current study represents an extension of the ELLF project. Participants who had previously submitted at least one house dust sample and lived approximately 100 km from the research center were invited to participate in home visits following their year 7 follow-up. In total, 50 participants were invited, 25 responded, and 24 agreed to participate. Home visits were conducted between February 2021 and July 2022, with each home visited twice—once in summer and once in winter. A total of 23 participants completed both seasonal visits, while one participant completed only the winter visit and was subsequently lost to follow-up. This resulted in 47 individual observations: 23 during summer visits and 24 during winter visits.

The Barwon Infant Study (BIS) is a pre-birth cohort comprising 1,074 infants, recruited using an unselected sampling frame to investigate the early-life origins of immune dysregulation in the modern environment (26). The study is based in the Barwon region of Victoria, Australia, which encompasses urban, rural, and coastal areas. Compared to Brisbane, the Barwon region has a cooler climate, with temperatures ranging from 5.2°C and 14.8°C in winter and 11.9°C to 25.0°C in summer (25). Participant characteristics broadly reflect those of the general Australian population, although there is a lower representation of families from non-English-speaking backgrounds. Further details are available in the cohort profile (26). Participants were followed up at multiple time points, including a home visit at 9 months of age for a sub-sample (*n* = 228), during which a household assessment was conducted and a dust sample was collected.

### 2.2 Air quality

#### 2.2.1 Direct measurements

During ELLF home visits, indoor and outdoor air quality were measured simultaneously. Particulate matter (PM) was measured with a TSI DustTrak™ DRX Aerosol monitor 8,533, while NO_2_ and CO were measured using a Dräger X-am 5,000 with a XXs NO_2_ LC and XXS CO LC sensor. The monitors were set up inside the home in a common area, with a second set placed outside in an area protected from rain. Where possible, sampling occurred over a 24-h period. Data were collected in 5-min intervals and averaged over the monitoring period.

#### 2.2.2 Modeled air pollution exposure

The BIS did not capture direct air quality measurements. These records were matched to two validated, satellite-based land-use regression models (27, 28) built for the Australian continent from spatial predictors, including land use and satellite information. The residential addresses of each child in both BIS and ELLF were geocoded and matched to an annual estimate of PM_2.5_ and NO_2_ to reflect long-term air pollution exposure.

### 2.3 Household characteristics survey

The ELLF cohort had previously completed an annual survey, which captured information on housing characteristics. We used expert opinion and previous research to determine the household characteristics and behaviors likely to influence indoor air quality and, thus, the presence of EPFRs. These relationships are depicted in a directed acyclic graph (DAG) (see Supplementary Figure S1). DAG is important in epidemiological research as it illustrates the assumed causal pathway between an exposure and an outcome and helps identify potential biases (29). A survey was developed for the ELLF home visits that matched questions asked in the BIS survey, with some additional questions identified by the DAG. A few examples of household characteristics collected were house age, type of heating system (e.g., gas, electric, wood), type of cooling system (e.g., air conditioning, fan) cooktop type, and the use of an extractor fan during cooking. Complete household characteristics are reported in Table 1. Participants completed the questionnaire online up to 1 week prior to each home visit. Responses were validated by study staff during each visit. In the BIS, the questionnaire was developed to allow pooling with international cohorts and included validated instruments where possible (26). A home visit review was conducted by a trained researcher, including a researcher-facilitated survey, and the research team validated responses.

**Table 1:** Descriptive results of EPFR concentration, air pollution, and season from the Early Life Lung Function (ELLF), Brisbane (*n* = 46) and Barwon Infant Study (BIS), Geelong, Australia (*n* = 178).

Variables	ELLF	BIS
Outcomes		
EPFR concentration (×10^17^ spins/g) median (IQR)	1.7 (3.6)	0.6 (1.2)
Air pollution and season		
24-h average indoor CO (ppm) mean (SD)	0.79 (0.853)	Not collected in BIS
24-h average indoor NO_2_ (μg/m^3^) mean (SD)	40.79 (9.62)	Not collected in BIS
24-h average indoor PM_2.5_ (μg/m^3^) mean (SD)	13.55 (13.19)	Not collected in BIS
24-h average ambient PM_2.5_ (μg/m^3^) mean (SD)	11.63 (6.30)	Not collected in BIS
Annual ambient PM_2.5_ (μg/m^3^) mean (SD)	6.13 (0.32)	7.25 (0.69)
Annual ambient NO_2_ (μg/m^3^) mean (SD)	12.25 (3.97)	5.36 (2.08)
Season of visit *n* (%)		
Summer	22 (47.8%)	31 (17.4%)
Winter	24 (52.2%)	43 (24.2%)
Autumn	Not collected in ELLF	71 (40.0%)
Spring	Not collected in ELLF	33 (18.5%)

We combined adjacent categories of categorical variables with low counts (≤ 4 in ELLF and ≤ 13 in BIS, corresponding to 10% of the sample size) and excluded binary variables with low counts (e.g., type of home (house vs. apartment): 43/46 in ELLF and 134/136 in BIS). In the ELLF cohort, the variables type of home, type of oven, and if any family member smokes or vapes were not able to be analyzed due to low counts; in the BIS cohort, the variables type of home, major renovations, use of mosquito coils, open-plan kitchen and heating in the child’s room were not analyzed for the same reason.

### 2.4 Dust collection

The primary outcome of this study was the presence of EPFRs in house dust. Dust samples were collected from household vacuums by the research team during home visits. Samples were frozen at −20°C in the laboratory of the Centre for Children’s Health Research, Brisbane, or Barwon Biomedical Research Laboratory, Geelong, prior to shipping to Louisiana State University for analysis. Dust samples were processed using a sieve shaker on a mesh size (<38 μm), in which radical concentrations were expressed in terms of spins per gram. The dust samples were analyzed using nano electron paramagnetic resonance (EPR) to determine spin concentrations, g-factor, and delta H peak-to-peak (ΔHp-p). (EMX nano, 2016). The correction for the content of oxygen-centered radicals (O-EFPRs) in the samples was based on the analysis of the EPFR spectra parameters, using a linear combination of the 3rd power of the g-tensor shift with respect to the position of the pure oxygen-centered radical (2.0049) and spectral broadening represented by the value of the delta H peak-to-peak. The resulting adjustment factor was then multiplied by the spin-concentration number. EPFRs were reported as the number of spins per gram of dust analyzed (spins/g) and analyzed as continuous variables. Samples with EPFR concentrations below the limit of detection (LOD) were imputed using half the lowest value of EPFR concentration for ELLF (*n* = 11) and BIS cohort (*n* = 46), respectively (30). Samples below the LOD for oxygen-centered environmentally persistent free radicals (O-EPFRs) were excluded from the analysis.

### 2.5 Statistical analysis

We used lasso-penalized regression to select predictors of EPFR and O-EPFR. We repeated 5-fold cross-validation 100 times and chose the lasso tuning parameter lambda (which determines the extent of the penalization) using percentile-lasso to stabilize our estimates against cross-validation variability, using the recommended 75^th^ percentile (31). We used unpenalized linear regression to obtain coefficient estimates and naive confidence intervals for the chosen variables and exact post-selection inference to obtain confidence intervals that account for the uncertainty in the variable selection (32, 33). For the ELLF cohort, in which up to two observations were available per household, we used generalized estimating equations with exchangeable correlation structures to account for the correlation between repeated measures. Although exact post-selection inference was the recommended approach for lasso selective inference in a recent review and simulation study, it does not account for the correlation across repeated measures and can produce confidence intervals that are too wide in high-dimensional data (low signal-to-noise) settings (33, 34).

In contrast, confidence intervals from linear regression on selected variables may be too narrow as they do not account for the uncertainty in the variable selection. We thus report two sets of confidence intervals: from linear regression on the selected variables and from post-selection inference. We used the R package glmnet for lasso regression (35) and the *selectiveInference* package for exact post-selection inference (32).

The outcome variable, EPFR concentration, was log-transformed, while pollutant variables—including 24-h average indoor and outdoor PM_2.5_, NO_2,_ and CO, as well as annual ambient PM_2.5_ and NO_2_—were log2-transformed. Indoor pollutant concentrations below the detection limit were imputed as half the detection limit.

Indoor-measured PM_2.5_, CO, and NO_2_ were included in the ELLF main analysis, whereas modeled ambient PM_2.5_ and NO_2_ were included for BIS. Sensitivity analysis was conducted on measured and modeled outdoor PM_2.5_, CO, and NO_2_ for ELLF. Random forest regression was used to examine whether our models identified a similar set of variables. Five-fold cross-validation was performed to ensure 100% of the data were used to build the random forest model. Random forest models with optimal hyperparameters were analyzed for variable importance (see Supplementary materials).

## 3 Results

**Figure 1:**
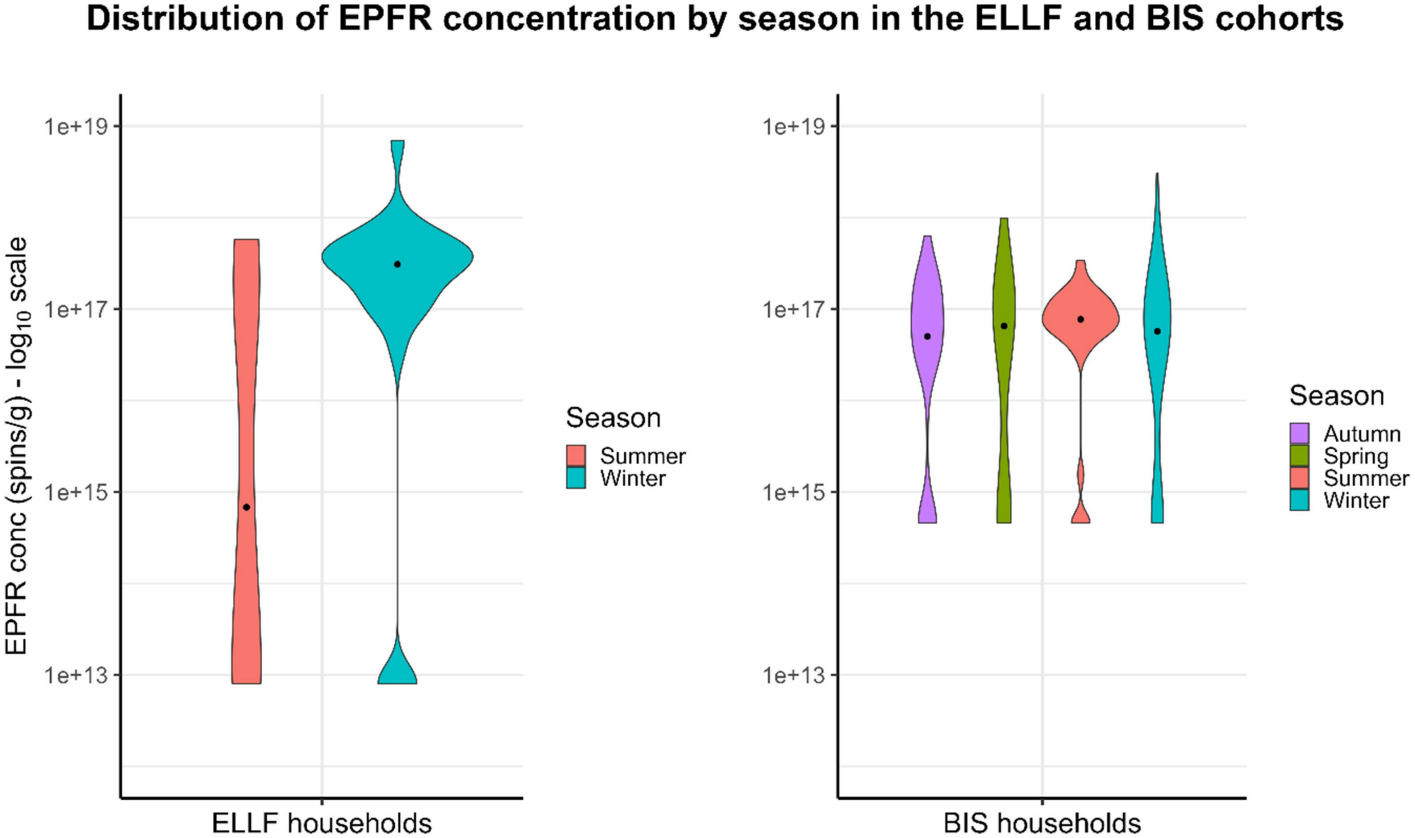
Distribution of EPFR concentration by season in the ELLF and BIS cohorts. EPFR concentration is presented on a logarithmic scale of base 10.

The EPFRs, air pollution, and household characteristics are shown in Tables 1, 2. The sample size for complete cases in the ELLF cohort was 46 homes, and in the BIS cohort, it was 136 homes. The median (interquartile range, IQR) EPFRs detected in the ELLF and BIS households were 1.65 × 10^17^ spins/g (IQR = 3.60 × 10^17^ spins/g) and 5.78 × 10^16^ spins/g (IQR = 1.21 × 10^17^ spins/g), respectively (Table 1). Based on the violin plots in Figure 1, the distribution of EPFR concentration in the ELLF cohort is much more spread out than in the BIS cohort, indicating greater variability in the Brisbane region. The EPFR concentration measured in both cohorts was below the low concentration group (< 4 × 10^17^ spins/g), as reported by Sly et al. (10), which found a protective association against wheezing in young children when compared to the high concentration group (≥ 6 × 10^17^ spins/g). Seasonal differences regarding EPFR concentration were inconsistent between the two geographical cohorts. In the ELLF cohort, lower median EPFRs were seen in warmer months of summer (7.06 × 10^14^ spins/g, IQR = 1.83 × 10^17^ spins/g) in comparison to colder months of winter (3.10 × 10^17^ spins/g, IQR = 3.13 × 10^17^ spins/g) (Figure 1). In the BIS cohort, there were slightly greater median EPFRs in summer (6.31 × 10^16^ spins/g, IQR = 4.53 × 10^16^ spins/g) when compared to winter (5.74 × 10^16^, IQR = 1.20 × 10^17^ spins/g) (Figure 1). However, the violin plots demonstrated longer upper tails in winter for both cohorts, indicating that the highest concentrations of EPFR tend to occur during colder weather.

**Table 2:** Descriptive results of household characteristics from the Early Life Lung Function (ELLF), Brisbane (*n* = 46), and Barwon Infant Study (BIS), Geelong, Australia (*n* = 178).

Household characteristics	ELLF	BIS
House physical structure		
Major house renovation in the previous 12 months *n* (%)		
Yes	7 (15.2%)	9 (5.1%)*
House outer wall material *n* (%)		
Brick	25 (54.3%)	123 (69.0%)
Weatherboard	21 (45.7%)	55 (31.0%)
Enclosed garage *n* (%)		
Yes	30 (65.2%)	137 (77.0%)
House age mean (SD)	43.72 (29.37)	34.70 (27.72)
Open plan kitchen *n* (%)		
Yes	40 (87.0%)	172 (96.6%)*
Heating, ventilation, and cooling		
Type of heating in living area *n* (%)		
Clean heating (electric, reverse-cycle air conditioner)	40 (87.0%)	110 (61.8%)
Dirty heating (gas, fireplace)	6 (13.0%)	68 (38.2%)
Type of heating in child’s room *n* (%)		
No heating	24 (52.2%)	2 (1.1%)*
Clean heating (electric, reverse-cycle air conditioner)	22 (47.8%)	176 (98.9%)*
Open windows/door in the past 7 days (days/week) mean (SD)	5.76 (2.13)	Not collected in BIS
Open windows/door in the past 7 days (hours/day) mean (SD)	Not collected in ELLF	6.10 (5.70)
Type of cooling in living area *n* (%)		
Clean cooling (air-conditioner)	28 (60.9%)	149 (83.7%)
Dirty cooling (ceiling/portable fan)	18 (39.1%)	29 (16.3%)
Type of cooling in child’s room *n* (%)		
Clean cooling (air-conditioner)	18 (39.1%)	95 (53.4%)
Dirty cooling (ceiling/portable fan)	28 (60.9%)	83 (46.6%)
Cooking appliances and practices		
Type of cooktop *n* (%)		
Electric (ceramic, coil, solid plate)	32 (69.6%)	24 (13.5%)
Gas	14 (30.4%)	154 (86.5%)
Frequency of extractor fan use *n* (%)		
Always	19 (41.3%)	73 (41.0%)
Occasionally	18 (39.1%)	82 (46.1%)
Never	9 (19.6%)	23 (12.9%)
Type of oven *n* (%)		
Electric	46 (91.3%)*	131 (73.6%)
Gas	4 (8.7%)*	47 (26.4%)
Indoor combustion activities		
Frequency of candle/incense use at home (days/year) mean (SD)	33.01 (80.52)	43.38 (86.4)
Mosquito coil use at home *n* (%)		
Yes	10 (21.7%)	6 (3.4%)*
Any family members smoke/vape *n* (%)		
Yes	3 (6.5%)*	22 (12.6%)
Total cigarette per day mean (SD)	Not collected in ELLF	1.56 (4.96)
Presence of indoor fireplace *n* (%)		
Yes	8 (17.4%)	26 (14.6%)
Traffic-related air pollution		
Neighborhood traffic *n* (%)		
Little to low	40 (87.0%)	103 (57.9%)
Some	0 (0%)	34 (19.1%)
High	6 (13%)	41 (23.0%)
Occupancy		
Household size* mean (SD)	4.22 (0.84)	Not collected in BIS
Presence of pets *n* (%)		Not collected in BIS
Yes	36 (78.3%)	
Flooring		
Carpeting in living area *n* (%)		
Yes	22 (47.8%)	Not collected in BIS
Carpeting in the kitchen or main area *n* (%)		
Yes	Not collected in ELLF	136(76.4%)
Carpeting in bedrooms *n* (%)		
Yes	Not collected in ELLF	161 (90.4%)
Fabric flooring in other areas *n* (%)		
Yes	Not collected in ELLF	101 (56.7%)
Cleaning practices		
Method to clean floors *n* (%)		
Mop/vacuum	39 (84.8%)	148 (83.1%)
Sweep	7 (15.2%)	30 (16.9%)
Method to clean surfaces *n* (%)		Not collected in BIS
Wet cloth	25 (54.3%)	
Dry cloth/dusting wand	21 (45.7%)	
Frequency of cleaning floors (times/week) mean (SD)	2.68 (2.88)	Not collected in BIS
Frequency of cleaning surfaces (times/week) mean (SD)	1.50 (1.50)	Not collected in BIS
Frequency of cleaning floors in the living area (days/year) mean (SD)	Not collected in ELLF	145.2 (115.5)
Frequency of cleaning floors in child’s room (days/year) mean (SD)	Not collected in ELLF	70.3 (55.4)

*Not included in the analysis due to low counts.

Annual air pollution exposure was similar for both cohorts. The average annual ambient PM_2.5_ concentration was 6.13 μg/m^3^ (SD = 0.32 μg/m^3^) in the ELLF cohort and 7.25 μg/m^3^ (SD = 0.69 μg/m^3^) in the BIS cohort. For annual average NO_2_, concentrations were 12.25 μg/m^3^ (SD = 3.97 μg/m^3^) in ELLF and 5.36 μg/m^3^ (SD = 2.08 μg/m^3^) in the BIS cohort. In the ELLF cohort, the monitored 24-h average PM_2.5_ concentrations were 13.55 μg/m^3^ indoors and 11.63 μg/m^3^ outdoors.

### 3.1 ELLF cohort

**Figure 2:**
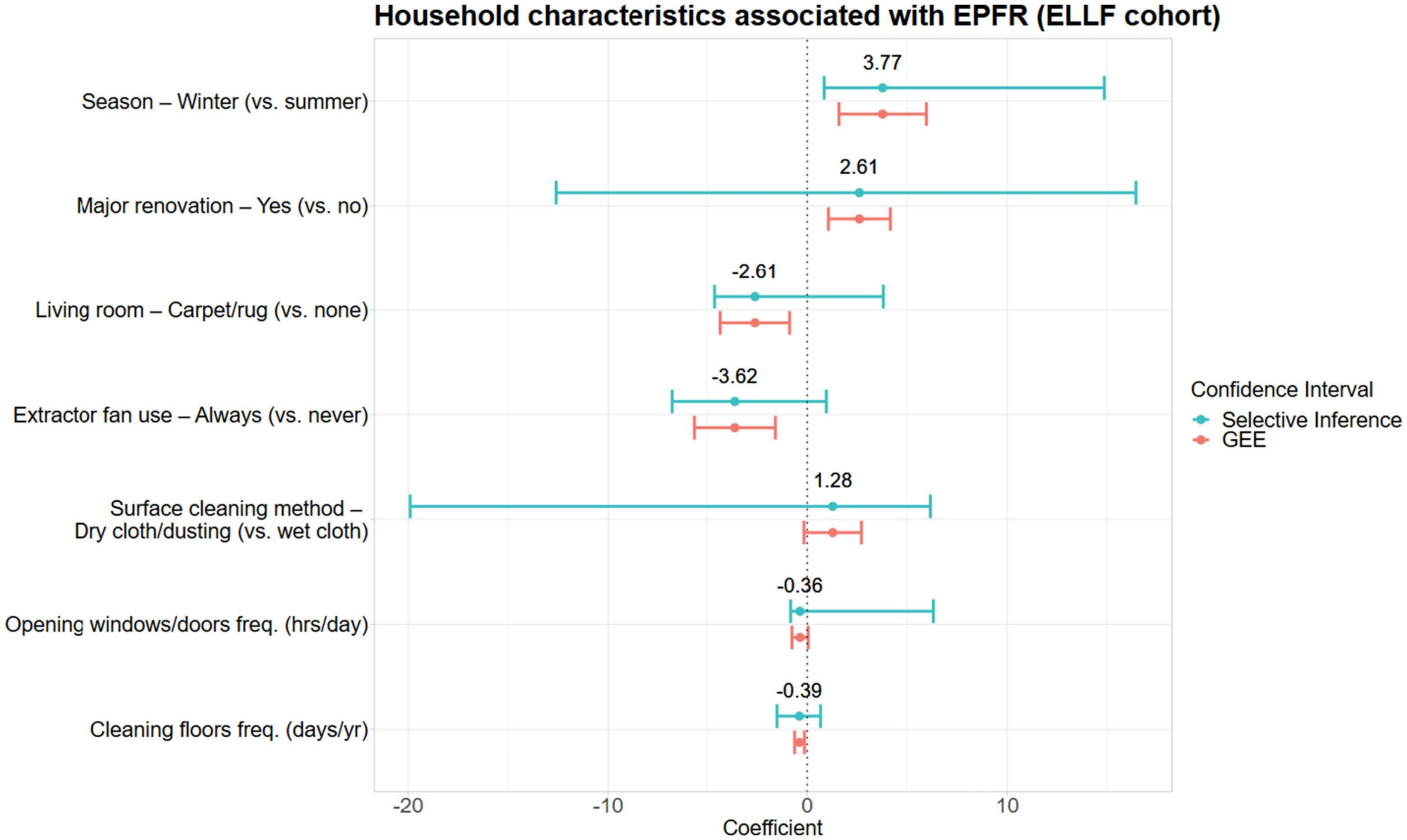
Household characteristics associated with EPFR concentrations in the ELLF cohort presented with a difference in mean log-EPFR concentration, β (log(spins/g)).

Seven household characteristics were associated with EPFR concentrations in house dust (Table 3 and Figure 2). The winter season was associated with the largest increase in EPFR concentration, followed by factors that affect dust re-suspension, such as using dry dusting methods to clean surfaces or major renovations to the home (Figure 2). Factors associated with lower household EPFR concentrations included always using an extractor fan during cooking, the presence of fabric flooring in the living area, more frequent floor cleaning, and more frequent opening of windows or doors (Figure 2). However, except for the winter season variable, post-selection inference revealed considerable uncertainty regarding the direction of these associations.

**Table 3:** Household characteristics associated with EPFR concentrations in the ELLF cohort.

Household characteristics	Difference in mean log-EPFR concentration, β (log(spins/g))	95% CI (Post-selection inference)	95% CI (Linear regression estimated by GEE on selected variables)
Season—Winter (vs. summer)	3.77	(0.91, 16.99)	(1.59, 5.96)
Major house renovation—Yes (vs. no)	2.61	(−12.68, 17.54)	(1.06, 4.16)
Living room—Carpet/rug (vs. no carpet/rug)	−2.61	(−4.69, 4.00)	(−4.35, −0.88)
Use of extractor fan—Always (vs. never)	−3.62	(−6.77, 1.78)	(−5.64, −1.59)
Method to clean surfaces—Dry cloth/dusting wand (vs. wet cloth)	1.28	(−21.59, 6.50)	(−0.16, 2.71)
Frequency of opening windows/doors (days/week)	−0.36	(−0.80, 7.60)	(−0.76, 0.05)
Frequency of cleaning floors (times/per week)	−0.39	(−1.71, 0.71)	(−0.63, −0.14)

The following household characteristics were not selected by the percentile lasso model: 24-h indoor concentrations of PM_2.5,_ CO, and NO_2_; type of heating and cooling in the living area and child’s room; use of candles/incense; cooktop type; housing material; garage type; house age; surface cleaning method and frequency; presence of pets; use of mosquito coils; occasional use of an extractor fan during cooking; household size; presence of a fireplace; and neighborhood traffic (see Supplementary Table S1).

### 3.2 BIS cohort

**Figure 3:**
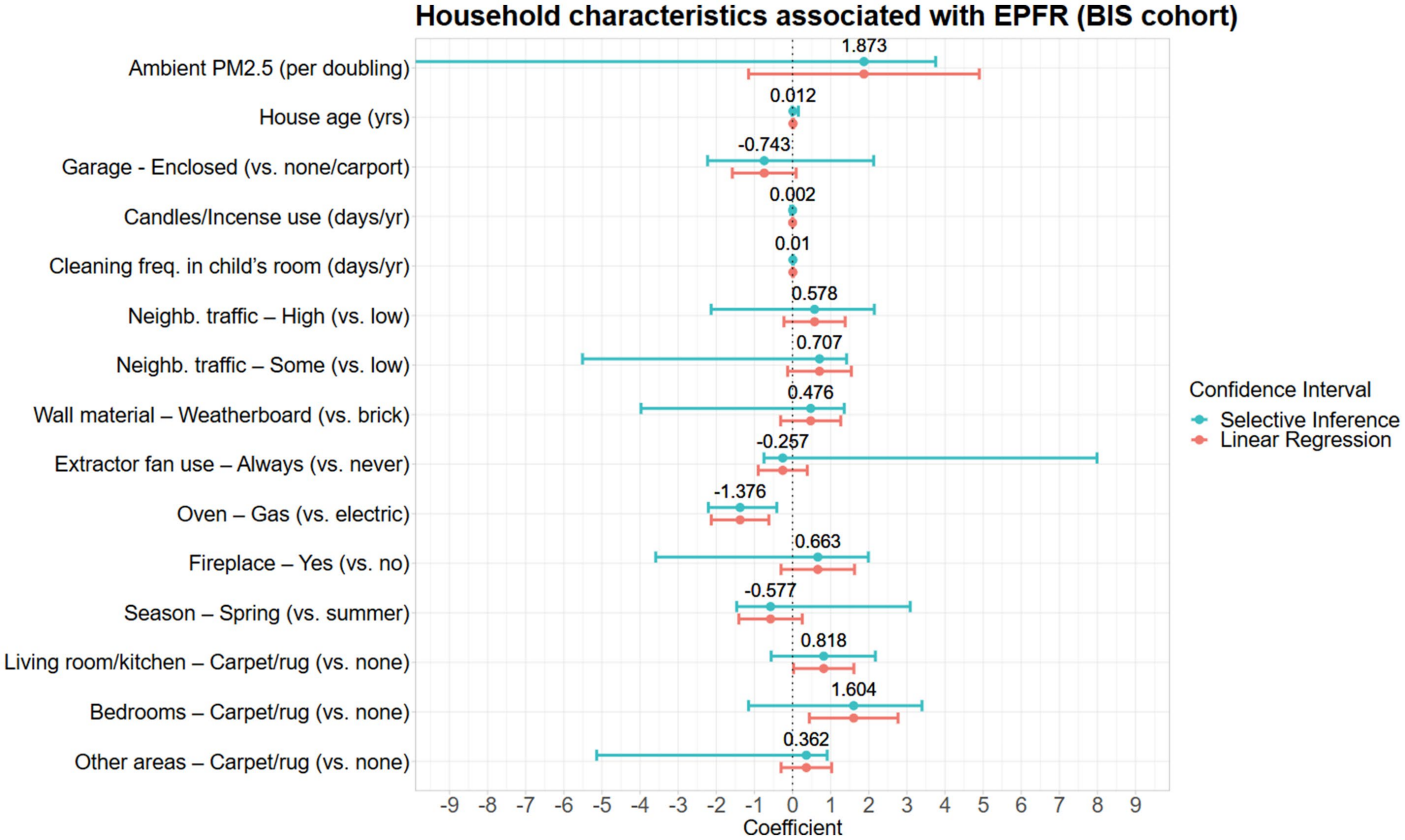
Household characteristics associated with EPFR concentrations in the BIS cohort presented with the difference in mean log-EPFR concentration, β (log(spins/g)).

A total of 15 household characteristics were associated with household EPFR concentrations (Table 4 and Figure 3). Factors associated with increased estimates of EPFR concentrations were annual ambient PM_2.5_, high and some neighborhood traffic, factors related to indoor combustion practices such as use of candles or incense and having a fireplace, age of house, weatherboard housing material, higher frequency of cleaning in the child’s room, and presence of carpet or rug in the house (Figure 3). Meanwhile, lower EPFR concentrations were associated with the spring season, always using the extractor fan during cooking, homes with enclosed garages, and gas ovens (Figure 3). However, with the exception of two variables (frequency of cleaning in the child’s room and type of oven), there was substantial uncertainty in the direction of the associations when we accounted for the uncertainty in the variable selection.

**Table 4:** Household characteristics associated with EPFR concentrations in the BIS cohort.

Household characteristics	Difference in mean log-EPFR concentration, β (log(spins/g))	95% CI (Post-selection inference)	95% CI (Linear regression on selected variables)
Annual ambient PM_2.5_ (per doubling)	1.87	(−50.59, 3.76)	(−1.15, 4.90)
House age (years)	0.01	(−0.01, 0.15)	(−0.004, 0.03)
Enclosed garage—Yes (vs. no)	−0.74	(−2.23, 2.13)	(−1.58, 0.09)
Candles/Incense usage (days/year)	0.002	(−0.05, 0.01)	(−0.001, 0.006)
Frequency of cleaning in child’s room (days/year)	0.01	(0.002, 0.016)	(0.004, 0.015)
Neighborhood traffic—High (vs. low)	0.58	(−2.14, 2.15)	(−0.22, 1.38)
Neighborhood traffic—Some (vs. low)	0.71	(−5.51, 1.42)	(−0.13, 1.54)
House outer wall material—Weatherboard (vs. brick)	0.48	(−3.97, 1.36)	(−0.31, 1.27)
Use of extractor fan—Always (vs. never)	−0.26	(−0.75, 7.99)	(−0.90, 0.39)
Type of oven—Gas (vs. electric)	−1.38	(−2.21, −0.41)	(−2.13, −0.62)
Fireplace—Yes (vs. no)	0.66	(−3.59, 1.99)	(−0.30, 1.63)
Season—Spring (vs. summer)	−0.58	(−1.47, 3.09)	(−1.41, 0.26)
Living room or kitchen—Carpet/rug (vs. no carpet/rug)	0.82	(−0.56, 2.17)	(0.03, 1.61)
Bedrooms—Carpet/rug (vs. no carpet/rug)	1.60	(−1.15, 3.40)	(0.44, 2.77)
Other areas—Carpet/rug (vs. no carpet/rug)	0.36	(−5.14, 0.91)	(−0.30, 1.02)

Household variables not chosen by the percentile lasso included annual ambient NO_2_, frequency of opening windows or doors, method and frequency of cleaning in the living room, type of cooktop, occasionally using the extractor fan during cooking, the heating method in the living room, cooling method in the living room and child’s bedroom, smoking or vaping, total number of cigarettes per day, and other seasons (autumn and winter) (see Supplementary Table S2).

### 3.3 BIS and ELLF cohort

**Figure 4:**
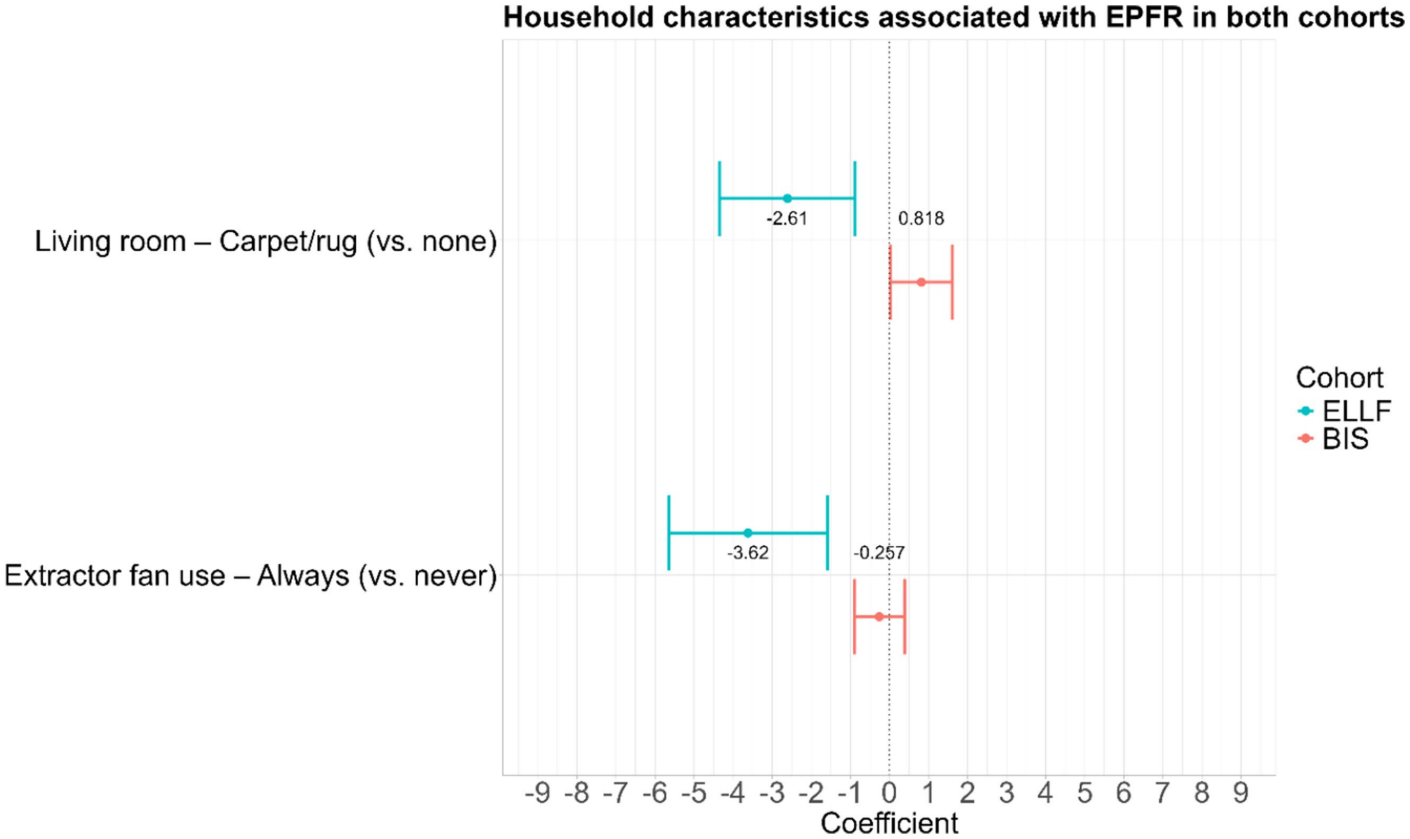
Household characteristics associated with EPFR in both cohorts presented with the difference in mean log-EPFR concentration, β (log(spins/g)). The 95% confidence intervals are GEE for ELLF and linear regression for BIS.

While the household characteristics collection differed slightly between the ELLF and BIS cohorts, two variables were found to be consistently associated with EPFR concentration in both cohorts: the use of an extractor fan and the presence of carpet/rug in the living room (Figure 4). Using an extractor fan during cooking consistently reduced EPFR concentration in both cohorts compared to never using one. However, the presence of a rug/carpet demonstrated an inconsistent direction of association of both cohorts, with the BIS cohort displaying an increase in EPFR concentration, while the ELLF cohort showed a decrease in EPFR concentration (Figure 4).

### 3.4 Sensitivity analysis

We examined the effect of 24-h monitored outdoor PM_2.5_ and NO_2,_ as well as CAND-modeled annual outdoor PM_2.5_ and NO_2_ in the ELLF cohort. Neither the short-term (24-h average) nor the annual ambient concentrations of particulate or gaseous pollutants were significantly associated with EPFRs or O-EPFRs (see Supplementary Tables S3–S6).

Household characteristics associated with O-EPFR concentration are included in Supplementary materials.

Random forest regression models showed that 17 out of the 28 household characteristics were chosen as important variables in predicting EPFR concentrations in the ELLF cohort, while 18 out of the 28 variables were chosen as important variables in the BIS cohort (see Supplementary Figure S2). In the ELLF cohort, all the variables chosen by lasso (Table 3) were also chosen by random forest. In the BIS cohort, several household characteristics, including some neighborhood traffic, always using an extractor fan during cooking, spring seasonal variation, and fabric flooring in other areas of the house, were chosen by lasso but were not chosen by random forest (see Supplementary Figure S3). However, the majority of the household characteristics chosen by lasso were also chosen by random forest (Table 4). To assess the robustness of model estimates, we conducted a non-parametric bootstrap analysis for the ELLF and BIS cohorts (see Supplementary Tables S7, S8).

## 4 Discussion

EPFRs are a relatively recent discovery, and research on residential exposure remains limited. To the best of our knowledge, the present study is the first to explore household characteristics associated with EPFR concentrations in house dust. In the ELLF study, we identified seven household characteristics or practices associated with EPFR concentrations, and in the BIS cohort, we identified 15 household characteristics. These household characteristics were indoor combustion activities, exposure to TRAP and ambient PM_2.5_ levels, seasonal variation, housing construction type, ventilation, and cleaning practices. Although we controlled for different variables in the two study regions, always using an extractor fan during cooking was consistently associated with a lower concentration of EPFRs in the home.

The lower EPFR concentrations observed in homes with consistent extractor fan usage during cooking highlight the significant impact of household cooking practices on indoor air quality. Research on cooking fume by-products has found that toxic compounds such as polycyclic aromatic hydrocarbons (PAH), aldehydes, alkanoic acids, heterocyclic aromatic amines, and other harmful products can be released when cooking at higher temperatures (36–38). It is established in the literature that EPFR concentrations can be generated from solid fuel combustion used for cooking (14, 20), but a close co-existence of EPFRs and cooking fume by-products, particularly PAHs, has also been reported (39–41). Regardless of whether EPFR originates from the fuel source used in cooking or from heating the food at high temperatures, the reduction of cooking emissions through regular use of an extractor appears to lower this oxidant component in house dust. It should be noted that the range of the confidence interval obtained indicates considerable uncertainty regarding the concise magnitude and direction of effects. These wide intervals may be due to limited statistical power. Further investigations with larger populations would help to validate and refine these estimates.

A systematic review found that major indoor PM_2.5_ sources were related to combustion activities, including cooking, smoking, wood stoves, and the use of candles and incense (42). Since studies have established the presence of EPFRs on PM surfaces, particularly PM_2.5_ generated from combustion activities (6, 7, 11, 12, 14), the use of candles, incense, gas stoves/ovens or fireplaces in homes could potentially increase EPFR concentrations in house dust. Our results from the BIS cohort support this hypothesis, demonstrating higher EPFR concentrations for homes with a fireplace and increased use of candles or incense. However, our results found that gas ovens were associated with lower EPFR concentrations than electric ovens in the BIS cohort. This association was contrary to prior assumptions, but this may be explained by interactions between EPFRs and other pollutants released during gas combustion rather than being solely attributed to PMs. A systematic review reported that indoor gas cooking and heating are found to be major sources of indoor NO_2_ (42), but the relationship between EPFRs and NO_2_ is still not yet established. One study reported that EPFRs were positively correlated with vehicle emissions on highways, particularly elemental carbon (EC) and NO_2_ (43). Another study supports the finding, demonstrating that PM_2.5_ exposure to NO_2_ increased EPFRs by 5–8 times, whereas PM_2.5_ exposure to NO and ozone did not change EPFR signals (44). Conversely, an experimental study demonstrated that EPFR concentrations were reduced from interacting with NO_2_ as EPFRs have the ability to donate electrons to form stable products (45). Additional research is needed to further understand the relationship between EPFRs and NO_2_ and to assess the impact of gas ovens on indoor air quality.

Xu et al. measured EPFR concentrations in PM_2.5_ released from various sources and found that PM_2.5_ from vehicle exhaust emissions generated the highest amount of EPFRs (22). The concentration of EPFRs was also observed to differ between agricultural areas and residential areas, with lower EPFR signals reported in agricultural areas due to lower vehicle emissions (16). The results of the present study demonstrated that annual ambient PM_2.5_ and neighborhoods with moderate to high traffic were associated with higher EPFR concentrations in the BIS cohort. Our findings on the importance of TRAP and ambient PM_2.5_ in house dust further suggest that outdoor infiltration significantly contributes to indoor air pollution.

We found the season to be important in both settings. In ELLF, a cohort in a sub-tropical region, EPFRs were higher in winter compared with summer. In BIS, a cool temperate region, EPFRs were lower in spring than summer after controlling for a different set of variables. A limitation of this study is that we did not have samples collected in all seasons in ELLF – study visits were only conducted in summer and winter. This finding was inconsistent with the literature, as previous studies have shown seasonal differences. For example, studies conducted in China found that EPFR concentrations were higher in colder months, with a study estimating an equivalent EPFR exposure approximating 23–73 cigarettes per day during the cold periods (12, 13, 20, 21, 46). The authors attribute this to the widespread use of high central heating, especially coal combustion, during winter. In addition, studies have identified that EPFR concentration with an adjacent oxygen atom was more prominent in colder weather, suggesting incomplete combustion (12, 20, 46). Coal combustion is not a heat source in Brisbane, so more work is needed to understand the potential sources in this setting. Rainfall may also influence seasonal variation in EPFR concentrations. The concentration of EPFRs associated with airborne PM_2.5_ in Beijing, China, was lower in the summer months, a result attributed to the clearing of PM_2.5_ by precipitation (13). Brisbane typically experiences higher rainfall during summer (47), whereas Geelong experiences its highest rainfall in spring and lowest rainfall during summer (48). These seasonal differences in precipitation across locations may play a role in the seasonal variation reported in the current study.

In the present study, housing construction type, garage type, house age, and housing material were associated with EPFRs in house dust. A systematic review reported that ventilation, house age, and attached garages are important household characteristics that predict indoor air pollutants in Canadian homes (49). These household structures likely influence the overall airtightness of a home (50–52). The air exchange rate (AER) is typically used to understand the leakiness of a house by determining the rate of outdoor air replacing indoor air in a specified space, and AER is one of the main factors that determine indoor air quality (42, 53). Measured AER > 1 (exchange per hour) indicates that outdoor sources are more pronounced, and indoor PM concentrations tend to closely resemble outdoor levels due to indoor air being replaced with outdoor air (54). An Australian report found older houses (over 50 years) in Australia tended to have higher AER (above the 90^th^ percentile of 1.3 h^−1^), signifying higher outdoor air penetration than newer houses (55). Further, houses built with weatherboard cladding (1.0 h^−1^) have higher mean AER values than brick houses (0.5 h^−1^) (55). Higher air penetration in timber-constructed houses may be due to an increased likelihood of structural degradation from termites, sunlight, and water damage (56). Furthermore, a study investigating air exchange rates (AERs) reported a mean AER of 0.44 h^−1^ with windows closed and 1.57 h^−1^ with windows open, highlighting the greater influence of outdoor air on the indoor environment when ventilation is increased (57). Our findings in the BIS cohort were consistent with those of (55), demonstrating higher EPFR concentrations of EPFRs in older homes and those constructed with weatherboard compared to brick. In the ELLF cohort, we found that higher natural ventilation and increased frequency of windows or doors opened in the ELLF cohort were associated with lower levels of EPFRs in house dust after controlling for ambient PM_2.5_ concentrations. High AER can either improve or worsen indoor air quality, depending on the location of the households and the levels of outdoor air pollution. The difference in EPFR concentration associated with high AER observed across different household characteristics in Brisbane and Geelong can be attributed to the lower annual outdoor PM_2.5_ levels in the ELLF cohort compared to the BIS cohort. Furthermore, the type of garage can influence indoor air quality, with previous research demonstrating that attached garages or underground parking garages led to higher concentrations of pollutants (volatile organic compounds, PM, CO, and NO_2_) inside the houses when compared to non-attached garages (42, 51, 52, 58). Pollutants generated in enclosed and attached garages from the car can accumulate and penetrate inside the home through walls and ceilings (51, 52). Therefore, we hypothesized that enclosed garages would be associated with greater EPFR concentrations due to greater levels of pollutants. However, we found a negative association in the BIS cohort. The reason for the observed negative association remains unclear, but we believe that the interaction between NO₂ and EPFRs, as previously discussed, may contribute.

Finally, measures to maintain cleanliness in the house were found to be important household characteristics of EPFRs in Australian homes. Measures such as removing shoes before entering homes, using doormats, removing carpets, dusting with damp cloths, and regular cleaning can reduce dust concentration and indoor air pollutants (57). We hypothesize that these cleaning measures can potentially lower EPFRs generated from indoor combustion or infiltration of outdoor air pollution. Our results showed that only the ELLF cohort demonstrated higher EPFRs for households that do not practice regular cleaning and use a dry cloth or dusting wand to clean surfaces. The presence of fabric flooring, carpets, or rugs throughout the home was associated with greater EPFR concentrations in the BIS cohort but lower EPFR concentrations in the living area for the ELLF cohort. The inconsistencies in the direction of associations between settings, with the BIS cohort demonstrating higher EPFR concentrations with increased cleaning frequency and fabric flooring throughout the home, may be attributed to ineffective cleaning practices. In addition, our findings demonstrated that houses that had undergone a major renovation were associated with higher EPFR concentrations in the ELLF cohort. Research on increased small PM (aerodynamic diameter of 1.7 μm) from house renovations (59) and EPFRs has not been extensively studied. The current understanding is that EPFRs are generated through combustion (6, 10–12, 14). Therefore, more research is warranted to understand the importance and mechanism of increased PM from major construction and EPFR concentrations.

While the results demonstrated that indoor EPFRs can be generated through household activities, components of TRAP—particularly PM_2.5_—also significantly influence EPFR concentrations within homes.

Our research provided insight into the potential impact of household characteristics on EPFR concentrations, which can potentially lead to adverse health effects. Future research should link our research findings on EPFRs—affected by factors such as the use of extractor fans during cooking, indoor combustion activities, exposure to TRAP and ambient PM2.5 levels, seasonal variation, housing construction type, ventilation, and cleaning practices—to their potential health effects.

This study has a few limitations that need to be acknowledged. Although the Barwon Infant Study (BIS) provided a valuable comparison cohort to the ELLF study, we were unable to age-match the children’s health outcomes, as dust samples in BIS were collected only at 9 months of age. As a result, our analysis focused solely on investigating household characteristics associated with EPFR concentrations in house dust across the two locations. Another limitation is the relatively small sample size, which constrained the statistical power of the study. Recruitment in both cohorts was limited by factors such as time, equipment availability, research staff, and funding limitations. Despite these challenges, the study also has several notable strengths. First, we anticipated that the small sample size in the ELLF cohort could lead to instability in model selection and wide confidence intervals. To address these concerns, we included data from the larger BIS cohort in Geelong. We also applied multiple approaches to evaluate the robustness of our results: (1) confidence intervals were estimated using both selective inference following lasso variable selection and standard linear models, and (2) we conducted a bootstrap analysis to assess the stability of results under resampling. These complementary methods allowed for a more comprehensive assessment of uncertainty (34). In addition, this prospective study reduced the risk of recall biases and provided more reliable information on exposures. Finally, the air pollution data for the ELLF cohort were obtained from monitors placed directly in participants’ homes, providing a more accurate representation of personal pollutant exposure compared to relying solely on publicly available environmental data.

## 5 Conclusion

This study found several household characteristics associated with EPFR levels in Australian homes, including the use of extractor fans during cooking, exposure to TRAP and ambient PM_2.5_ levels, indoor combustion activities, seasonal variations, housing construction type, ventilation, and cleaning practices. Environmentally Persistent Free Radicals (EPFRs) are oxidant components of air pollution and may play a role in human health impacts of air pollution exposure. This study found EPFRs are present in house dust and that key factors influencing EPFR concentrations included the usage of extractor fans while cooking, traffic-related air pollution, ambient PM_2.5_, indoor combustion activities, season, housing structures, ventilation, and cleaning habits. Notably, consistent use of extractor fans while cooking was associated with lower EPFR concentrations in household dust. These findings underscore the importance of household practices and environmental factors in determining indoor air quality and the persistence of pollutants within the home.

## Data Availability

The datasets presented in this article are not readily available because data is only available on application to cohorts. Requests to access the datasets should be directed to ellf@uq.edu.au, bis@barwonhealth.org.au.
