# Supplementary material for: Associations between household characteristics and environmentally persistent free radicals in house dust from two Australian locations
Source: Front Public Health. 2025 Jun 18;13:1603114. doi: 10.3389/fpubh.2025.1603114 (PMC12213907; doi:10.3389/fpubh.2025.1603114)
Supplement: Supplementary file 1 [file Table_1.docx]

# **Supplementary Materials**

**Household characteristics associated with environmentally persistent free radicals in house dust in two Australian locations**

Wen Ray Lee^1^, Prakash Dangal^2^, Gaurav Langan^1^, Nina Lazarevic^3^, Zhiwei Xu^4^, Stephania A. Cormier^2^, Slawo Lomnicki^2^, Peter D Sly^1^, Dwan Vilcins^1^

^1^The University of Queensland, Child Health Research Centre, Children’s Health and Environment Program, South Brisbane, QLD 4101, Australia.

^2^Superfund Research Centre, Louisiana State University, Baton Rouge, United States.

^3^ National Centre for Epidemiology and Population Health, Australian National University, Canberra, ACT 2601, Australia.

^4^School of Medicine and Dentistry, Griffith University, Gold Coast, QLD 4215, Australia.

**Corresponding author**: Dwan Vilcins | Email: [d.vilcins@uq.edu.au](mailto:d.vilcins@uq.edu.au)


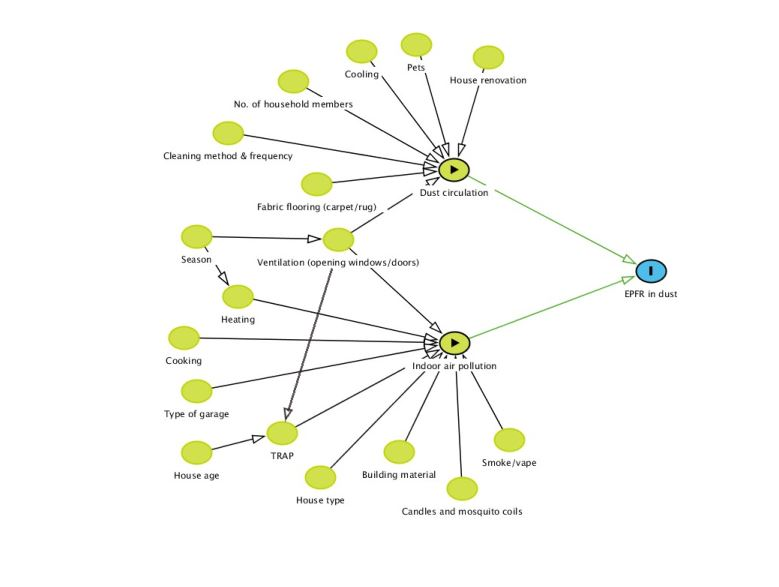


Figure S1 Directed Acyclic Graph of household characteristics and presence of EPFR in dust.

Table S1 Frequency of important variables in the 100 cross-validation LASSO models for ELLF cohort (Indoor PM_2.5_).

| **Household characteristics** | **EPFR** | **O-EPFR** |
| --- | --- | --- |
| Season – Winter (vs. summer)  24-hour average indoor PM_2.5_ (per doubling) | 100  0 | 99  0 |
| Major house renovation – Yes (vs. no)  Living room – Carpet/rug (vs. no carpet/rug)  Heating in living room – Dirty (vs. clean)  Heating in child’s room – Clean (vs. no heating)  Cooling in living room – Dirty (vs. clean)  Cooling in child’s room – Dirty (vs. clean)  Use of candles/incense (days/year) | 96  100  61  1  0  1  0 | 85  65  -  55  85  3  4 |
| Use of mosquito coil – Yes (vs. no)  Type of cooktop – Gas (vs. electric)  Use of extractor fan – Always (vs. never)  Use of extractor fan – Occasionally (vs. never)  Housing material – Weatherboard (vs. brick) | 0  0  100  0  0 | 82  0  71  0  55 |
| Enclosed garage – Yes (vs. no)  Age of house (years)  Household size (total number of family members)  Frequency of windows/doors opened (days/week)  Method of cleaning – Sweep (vs. mop/vacuum) | 6  6  50  83  0 | 82  0  95  36  0 |
| Method of cleaning surface – Dry cloth/dusting wand (vs. wet cloth)  Pets – Yes (vs. no)  Open plan kitchen – Yes (vs. no)  Fireplace – Yes (vs. no)  Neighbourhood traffic – Low (vs. high)  Frequency of cleaning floors (times/week)  Frequency of cleaning surfaces (times/week)  24-hour average indoor CO (per doubling)  24-hour average indoor NO_2_ (per doubling) | 96  0  61  0  3  100  0  0  49 | 85  13  0  0  36  81  85  0  85 |

Table S2 Frequency of important variables in the 100 cross-validation LASSO models for BIS cohort (Ambient PM_2.5_).

| **Household characteristics** | **EPFR** | **O-EPFR** |
| --- | --- | --- |
| Annual ambient NO_2_ (per doubling)  Annual ambient PM_2.5_ (per doubling) | 67  87 | 0  26 |
| House age (years)  Enclosed garage – Yes (vs. no)  Other areas – Carpet/rug (vs. no carpet/rug)  Frequency of windows opened (hours/day)  Use of candles/incense (days/year)  Method of cleaning floors – Sweep (vs. mop/vacuum)  Frequency of cleaning living room (days/year) | 100  95  90  1  87  57  54 | 0  0  0  0  0  0  0 |
| Frequency of cleaning child’s room (days/year)  Neighbourhood traffic – High (vs. low)  Neighbourhood traffic – Some (vs. low)  Housing material – Weatherboard (vs. brick)  Type of cooktop – Gas (vs. electric)  Use of extractor fan – Always (vs. never)  Use of extractor fan – Occasionally (vs. never) | 100  99  87  95  1  90  57 | 3  51  4  1  1  1  1 |
| Type of oven – Gas (vs. electric)  Heating in living room – Dirty (vs. clean)  Cooling in living room – Dirty (vs. clean)  Cooling in child’s room – Dirty (vs. clean)  Smoking/vaping – Yes (vs. no)  Total number of smokes (per day)  Fireplace – Yes (vs. no) | 100  1  3  29  57  1  97 | 3  0  3  0  2  0  2 |
| Season – Autumn (vs. summer)  Season – Spring (vs. summer)  Season – Winter (vs. summer)  Living room or kitchen – Carpet/rug (vs. no carpet/rug)  Bedrooms – Carpet/rug (vs. no carpet/rug) | 44  95  22  100  100 | 0  0  0  3  13 |

Table S3 Important household characteristics identified in the LASSO regression model in the ELLF cohort for O-EPFR Concentration (24-hour ambient).

| **Household characteristics** | **Exponential change in mean EPFR concentration** | | **95% CI (Post-selection inference)** | **95% CI (GEE)** |
| --- | --- | --- | --- | --- |
| Season – Winter (vs. summer) | 3.21 | (-0.50, 6.09) | | (1.71, 4.72) |
| Major house renovation – Yes (vs. no)  Cooling in living room – Dirty (vs. clean) | 1.92  0.77 | (-9.36, 12.52)  (-16.03, 11.63) | | (0.67, 3.18)  (0.16, 1.37) |
| Use of mosquito coil – Yes (vs. no) | 0.38 | (-19.29, 4.96) | | (-0.30, 1.07) |
| Enclosed garage – Yes (vs. no)  Household size (total number of family members) | 1.17  1.06 | (-17.84, 8.17)  (-1.74, 4.49) | | (0.05, 2.28)  (0.30, 1.82) |
| Method of cleaning surfaces – Dry cloth/dusting wand (vs. wet cloth)  Frequency of cleaning floors (times/week)  Frequency of cleaning surfaces (times/week)  24-hour ambient NO_2_ (per doubling) | -0.04  -0.48  -0.04  -0.02 | (-13.83, 5.99)  (-2.29, 4.24)  (-2.48, 3.96)  (-10.49, 21.33) | | (-1.03, 0.94)  (-0.85, -0.10)  (-0.34, 0.26)  (-2.64, 2.60) |

Table S4 Frequency of important variables in the 100 cross-validation LASSO models for ELLF cohort (24-hour ambient).

| **Household characteristics** | | | **EPFR** | **O-EPFR** |
| --- | --- | --- | --- | --- |
| Season – Winter (vs. summer)  24-hour average ambient PM_2.5_ (per doubling) | 100  0 | | | 99  0 |
| Major house renovation – Yes (vs. no)_  Living room – Carpet/rug (vs. no carpet/rug)  Heating in living room – Dirty (vs. clean)  Heating in child’s room – Clean (vs. no heating)  Cooling in living room – Dirty (vs. clean)  Cooling in child’s room – Dirty (vs. clean)  Use of candles/incense (days per year) | | | 97  100  61  1  0  1  0 | 84  64  0  54  84  0  1 |
| Use of mosquito coil – Yes (vs. no)  Type of cooktop – Gas (vs. electric)  Use of extractor fan – Always (vs. never)  Use of extractor fan – Occasionally (vs. never)  Housing material – Weatherboard (vs. brick) | | 0  0  100  0  0 | | 81  0  69  0  54 |
| Enclosed garage – Yes (vs. no)  Age of house (years)  Household size (total number of family members)  Frequency of windows/doors opened (days/week)  Method of cleaning floors – Sweep (vs. mop/vacuum) | | 6  6  51  86  0 | | 81  0  94  32  0 |
| Method of cleaning surface – Dry cloth/dusting wand (vs. wet cloth)  Pets – Yes (vs. no)  Open plan kitchen – Yes (vs. no)  Fireplace – Yes (vs. no)  Neighbourhood traffic – Low (vs. high)  Frequency of cleaning floors (times/week)  Frequency of cleaning surfaces (times/week)  24-hour average ambient CO (per doubling)  24-hour average ambient NO_2_ (per doubling) | | 97  0  61  0  3  100  0  0  51 | | 84  12  0  0  32  83  84  0  84 |

Table S5 Important household characteristics identified in the LASSO regression model in the ELLF cohort for O-EPFR concentration (annual ambient).

| **Household characteristics** | **Exponential change in mean O-EPFR concentration** | **95% CI (Post-selection inference)** | **95% CI (Generalized Estimating Equations)** |
| --- | --- | --- | --- |
| Season – Winter (vs. summer) | 2.00 | (-9.23, 21.51) | (-0.09, 4.10) |
| Major house renovation – Yes (vs. no)  Heating in child’s room – Clean (vs. no heating) | 2.91  -1.47 | (-7.34, 15.17)  (-11.40, 13.38) | (1.00, 4.82)  (-3.34, 0.41) |
| Cooling in living area – Dirty (vs. clean) | 1.79 | (-16.36, 16.30) | (-0.31, 3.89) |
| Use of mosquito coil – Yes (vs. no) | 1.17 | (-4.48, 3.32) | (-0.09, 2.43) |
| Use of extractor fan – Always (vs. never)  Household size (total number of family members)  Frequency of opening windows/doors (days/week)  Pets – Yes (vs. no) | 0.15  1.38  -0.04  1.18 | (-13.03, 6.96)  (-1.55, 7.76)  (-1.91, 4.05)  (-6.25, 5.49) | (-1.57, 1.87)  (0.37, 2.39)  (-0.42, 0.34)  (-0.20, 2.56) |
| 24-hour average ambient CO (per doubling) | -0.004 | (-3.74, 13.02) | (-0.95, 0.94) |

Table S6 Frequency of important variables in the 100 cross-validation LASSO models for ELLF cohort (annual ambient).

| **Household characteristics** | **EPFR** | **O-EPFR** |
| --- | --- | --- |
| Season – Winter (vs. summer)  Annual ambient PM_2.5_ | 7  0 | 99  0 |
| Major house renovation – Yes (vs. no)_  Living room – Carpet/rug (vs. no carpet/rug)  Heating in living room – Dirty (vs. clean)  Heating in child’s room – Clean (vs. no heating)  Cooling in living room – Dirty (vs. clean)  Cooling in child’s room – Dirty (vs. clean)  Use of candles/incense (days per year) | 0  0  -  0  0  0  0 | 84  1  -  78  77  62  9 |
| Use of mosquito coil – Yes (vs. no)  Type of cooktop – Gas (vs. electric)  Use of extractor fan – Always (vs. never)  Use of extractor fan – Occasionally (vs. never)  Housing material – Weatherboard (vs. brick) | 0  0  0  0  0 | 79  4  82  1  14 |
| Enclosed garage – Yes (vs. no)  Age of house (years)  Household size (total number of family members)  Frequency of windows/doors opened (days/week)  Method of cleaning floors – Sweep (vs. mop/vacuum) | 0  0  0  0  0 | 16  1  100  79  7 |
| Method of cleaning surfaces – Dry cloth/dusting wand (vs. wet cloth)  Pets – Yes (vs. no)  Open plan kitchen – Yes (vs. no)  Fireplace – Yes (vs. no)  Neighbourhood traffic – Low (vs. high)  Frequency of cleaning floors (times/week)  Frequency of cleaning surfaces (times/week)  24-hour average ambient CO (per doubling)  Annual ambient NO_2_ (per doubling) | 0  0  0  0  -  7  0  7  0 | 0  78  0  -  -  19  6  83  1 |


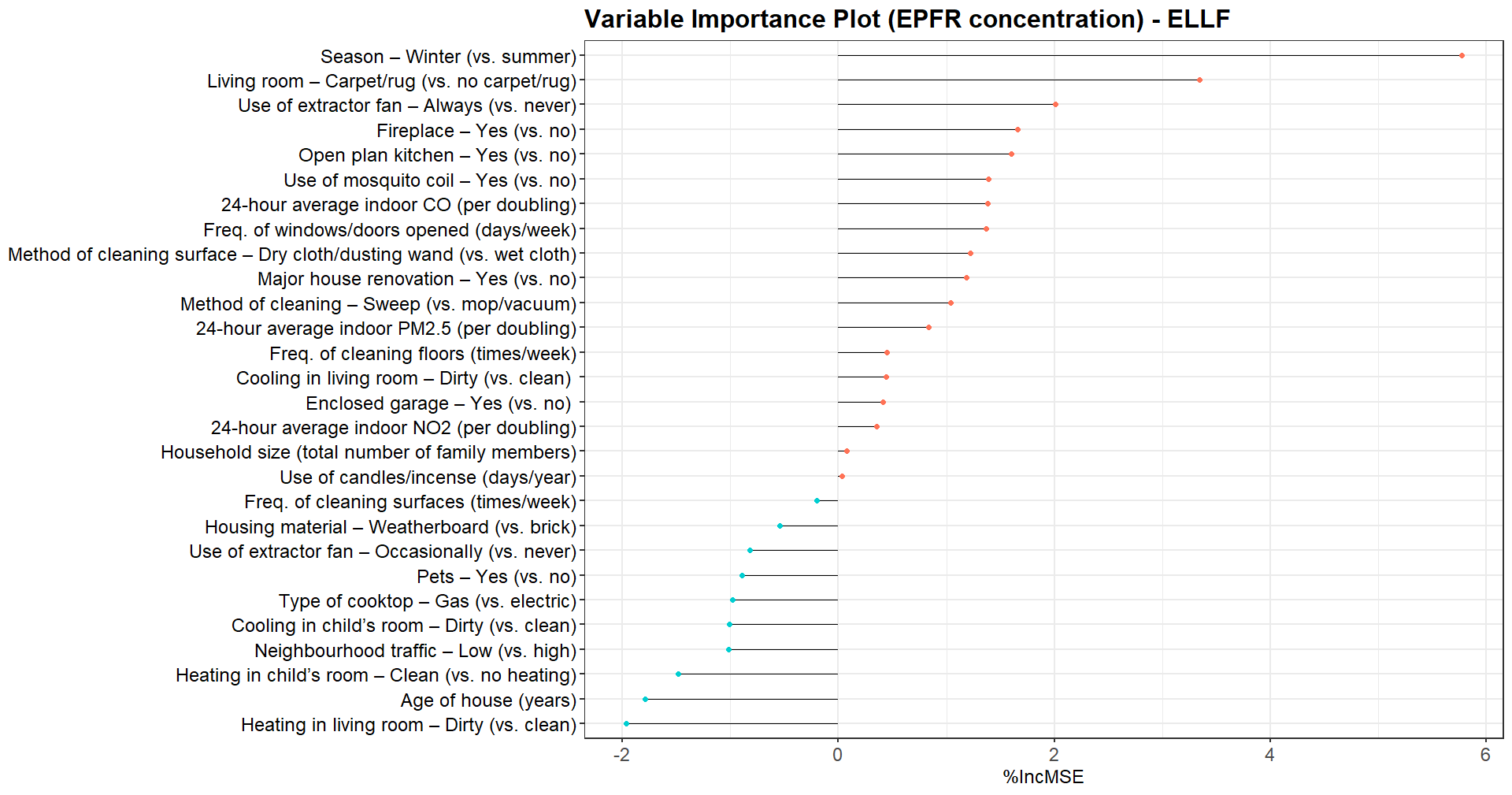


Figure S2 Random Forest Variable Importance Plot – from most important (red point) to least important (blue point) household characteristic of EPFR concentration in the ELLF cohort (included 24-hour average indoor PM2.5, NO2 and CO).


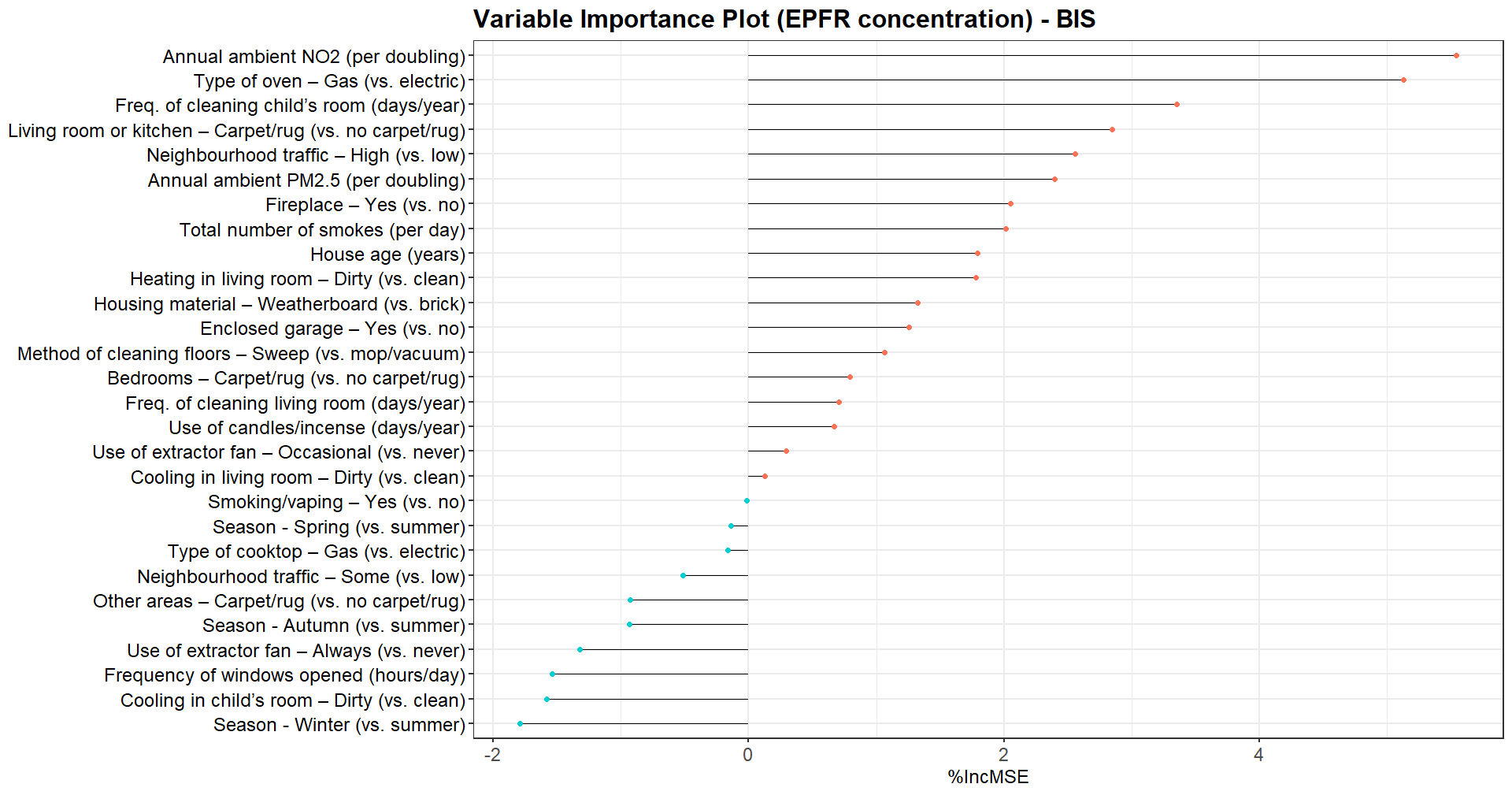


Figure S3 Random Forest Variable Importance Plot – from most important (red point) to least important (blue point) household characteristic of EPFR concentration in the BIS cohort (included ambient PM_2.5_ and NO_2_).

Table S7 Household characteristics associated with EPFR concentrations in the ELLF cohort (based on 1000 Bootstrap samples).

| **Household characteristics** | **Difference in mean**  **log-EPFR concentration,**  **β (log(spins/g))** | **95% CI (Bootstrap)** |
| --- | --- | --- |
| Season – Winter (vs. summer) | 3.64 | (1.23, 6.12) |
| Major house renovation – Yes (vs. no) | 2.69 | (-0.37, 6.00) |
| Living room – Carpet/rug (vs. no carpet/rug) | -2.06 | (-4.20, 0.16) |
| Use of extractor fan – Always (vs. never) | -3.89 | (-6.42, -1.31) |
| Method to clean surfaces – Dry cloth/dusting wand (vs. wet cloth) | 1.13 | (-0.98, 0.25) |
| Frequency of opening windows/doors (days/week) | -0.39 | (-1.01, 3.32) |
| Frequency of cleaning floors (times/per week) | -0.35 | (-0.68, 0.21) |

Table S8 Household characteristics associated with EPFR concentrations in the BIS cohort (based on 1000 Bootstrap samples)

| **Household characteristics** | **Difference in mean**  **log-EPFR concentration,**  **β (log(spins/g))** | **95% CI (Bootstrap)** |
| --- | --- | --- |
| Annual ambient PM_2.5_ (per doubling) | 1.95 | (-1.09, 5.21) |
| House age (years) | 0.01 | (-0.003, 0.03) |
| Enclosed garage – Yes (vs. no) | -0.74 | (-1.54, 0.04) |
| Candles/Incense usage (days/year) | 0.003 | (-0.0006, 0.006) |
| Frequency of cleaning in child’s room (days/year) | 0.01 | (0.004, 0.014) |
| Neighbourhood traffic – High (vs. low) | 0.54 | (-0.31, 1.36) |
| Neighbourhood traffic – Some (vs. low) | 0.70 | (0.02, 1.41) |
| Housing material – Weatherboard (vs. brick) | 0.49 | (-0.29, 1.28) |
| Use of extractor fan – Always (vs. never) | -0.24 | (-0.86, 0.42) |
| Type of oven – Gas (vs. electric) | -1.38 | (-2.15, -0.62) |
| Fireplace – Yes (vs. no) | 0.67 | (-0.21, 1.54) |
| Season – Spring (vs. summer) | -0.57 | (-1.41, 0.22) |
| Living room or kitchen – Carpet/rug (vs. no carpet/rug) | 0.79 | (-0.04, 1.60) |
| Bedrooms – Carpet/rug (vs. no carpet/rug) | 1.64 | (0.41, 2.94) |
| Other areas – Carpet/rug (vs. no carpet/rug) | 0.34 | (-0.35, 1.03) |
